# The prevalence of smartphone addiction and its related risk factors among Palestinian high school students: a cross-sectional study

**DOI:** 10.3389/fpsyt.2025.1636080

**Published:** 2025-09-24

**Authors:** Muna Ahmead, Etaf Maqboul, Eman Alshawish, Mohammad Dweib

**Affiliations:** ^1^ Faculty of Public Health, Al-Quds University, Jerusalem, Palestine; ^2^ Faculty of Nursing & Health Sciences – Departments, Bethlehem University, Bethlehem, Palestine; ^3^ Faculty of Nursing, An-Najah National University, Nablus, Palestine

**Keywords:** smartphone, depression, anxiety, social support, addiction, Palestine

## Abstract

**Background:**

Smartphone addiction is a significant social and health problem. There is limited research on smartphone addiction in Palestine. The current study aimed to assess the prevalence of smartphone addiction and its association with sociodemographic variables, depression, anxiety, and social support among 12th grade students.

**Methods:**

The study utilized a cross-sectional research design. A self-reported questionnaire, including the Smartphone Addiction Scale (SPAS), the OSLO Social Support Scale, and the Hospital Anxiety and Depression Scale (HADS), was used to gather data.

**Results:**

A total of 1,083 participants were recruited, of whom 27.3% had depression, 50.5% had anxiety, 57.3% experienced smartphone addiction, and 17.6% had strong social support. The study found that students with weak or moderate teacher relationships (AOR: 2.854, p < 0.001), disrupted sleep with smartphones (AOR: 2.143, p < 0.001), negative impact of smartphone usage on studies (AOR: 3.016, p < 0.001), and poor or weak social support (AOR: 3.051, p < 0.001) were at risk of smartphone addiction. Participants who reported no impact on their sleep time (AOR: 0.478, p-value = 0.001), used smartphones for less than 2 hours daily (AOR: 0.347, p < 0.001), and used smartphones for 2 to 3 hours daily (AOR: 0.684, p = 0.037) were less likely to develop smartphone addiction.

**Conclusion:**

Smartphone addiction was prevalent in high school students in this study. Weak teacher relationships, sleep disturbances, negative academic effects, and insufficient social support may lead to smartphone addiction. Programs that educate students, parents, and educators on smartphone addiction can prevent it and help detect and manage smartphone use problems.

## 1 Introduction

Schoolchildren are among the age groups most influenced by communication technology (1). They are particularly interested in purchasing smartphones, as they spend a considerable amount of time on these devices, which can affect their relationships with others and their learning (2, 3). The number of smartphone users has increased substantially. Between 2024 and 2029, the worldwide number of smartphone users is predicted to climb by 1.8 billion (4). In 2023, 78% of people aged 10 and up globally own mobile phones, and more than three-quarters of the world’s population does (5). In 2021, almost 90% of 14- to 18-year-olds owned smartphones (6). Smartphones positively impact communication and information dissemination, as well as the exchange of useful experiences between nations through their many uses (7). Moreover, smartphones enhance daily life by offering varied social statuses and identities for youth and mitigating deficiencies in social interactions (1). The rapid proliferation of these devices is ascribed to their portability and capacity to offer diverse functionalities, including Internet browsing, email access, social media connectivity, real-time broadcasting, photography, navigation, and multimedia playback (8). Consequently, smartphones have become essential in everyday life owing to their many capabilities for information acquisition, communication, education, and entertainment (9, 10).

One major risk factor for this age group is smartphone addiction, which is more common among younger individuals because of the extensive use of smartphones among them (11). Research has shown that when smartphone use interferes with tasks that are part of everyday living, it can lead to behavior that is problematic or addictive (8, 12). Addiction to smartphones was found in 39% to 44% of Indian teenagers (13). Also, 11.4% of South Korean teenagers were addicted to smartphones, with 2.2% of that group reporting that their addiction was interfering with their daily lives (11). Further, 15% of American persons in the 18–29 group were considered to have a serious smartphone addiction (14). Smartphone addiction has several terms of reference, including “nomophobia,” “problematic smartphone use,” and “smartphone dependence.” (15). According to the American Psychological Association (APA), addiction is “a condition of psychological and/or physical reliance on the consumption of drugs or other substances, including alcohol, or on activities or behaviors, such as sexual activity, exercise, and gambling (16).” Smartphone addiction is defined as a problem that affects users’ daily lives, with clinical symptoms including decreased concentration, tolerance, and control; mood problems; and withdrawal symptoms (17). Smartphone addiction is characterized as frequent smartphone use for social networking and entertainment, short intervals between the last use before sleep and the first use upon waking, and excessive daily usage (10, 18). Excessive smartphone usage can affect individuals’ physical, mental, and social well-being, as well as their educational and professional lives (19). There are many possible dangers linked to smartphone use and addiction, such as feeling tired, increased stress, headaches, trouble focusing, feeling depressed, experiencing loneliness and weaker relationships, not getting enough sleep, and poor school performance (20–24). Researchers have found that young people who use their phones a lot are more likely to develop phobias related to their devices. These phobias include behaviors like sleeping next to the phone, constantly checking for messages and calls, being anxious in places without network coverage or with limited access, and constantly keeping the phone powered on (25). Students’ problems with smartphone addiction harm their ability to communicate, socialize, and achieve success in school (8, 13, 26).

Since 1967, Israeli forces have occupied the West Bank and Gaza Strip (27). The Israeli-Palestinian conflict’s persistence for decades has severely affected the health of Palestinian youth and others (28). Recently, Palestine’s political and humanitarian situations have dramatically worsened due to a new conflict that broke out on October 7, 2023, when Hamas initiated an attack. After that, Israel launched a military operation on Gaza (27). As of July 23, 2025, Israel’s military invasion in Gaza has resulted in the deaths of over 59,219 Palestinians and injured an additional 143,045 (29). Many civilians are living in poor conditions due to a lack of basic necessities, such as food, electricity, medical treatment, and humanitarian assistance. Children have missed out on an entire year of education and numerous bombings targeted schools housing displaced families (30). Additionally, the West Bank has experienced an increase in deaths, extensive devastation, and forced relocation as a result of the violations committed by Israeli forces and settler attacks (31). There were 806 Palestinian deaths in the West Bank as of January 2025 (32). In addition, the Israeli military enforces movement restrictions between cities in the West Bank (31). There is significant evidence that the mental health of young Palestinians in the West Bank is negatively impacted by exposure to violence and indirect stressors related to the conflict, such as economic hardship, mobility restrictions and mental problems such as depression, anxiety and posttraumatic stress disorder (PTSD) (27, 28, 33). To cope with tension, anxiety, academic demands, adoration, separations, and other challenges, adolescents are more prone to use smartphones as a coping method (34).

In Palestine, 97% of households have at least one mobile line. Approximately 97% of people aged 18 to 29 use the Internet, including 99% in the West Bank and 92% in the Gaza Strip. According to statistics, 93% of young people own a smartphone, including 99% in the West Bank and 83% in the Gaza Strip (35). Nonetheless, there is a lack of studies on smartphone addiction among the younger generation, particularly twelfth-grade students. This educational period is regarded as crucial to students and their families. The Palestinian education system is structured in a 12-year process that includes grades 1-12. After completing 12 years of formal education, twelfth-grade students have to pass the Tawjihi (General Secondary Education Certificate test), a national test conducted by Palestine’s Ministry of Education and Higher Education. This examination is an important benchmark in the Palestinian educational system. It serves as a systematic appraisal of students’ qualifications for further education and potential job paths. As a result, students face enormous pressure from teachers and families to study extensively to get high test scores. They are more likely to develop anxiety symptoms such as excessive worry, irritability, concentration difficulties, depression, and thoughts of suicide (36). Additionally, students have used their smartphones for online learning recently because of school closures caused by the COVID-19 epidemic and continuous political violence and war in Palestine. There is a lack of research on the negative effects of smartphone usage among 12th grade students. The purpose of this study was to assess the prevalence of smartphone addiction among 12th grade students. The study also sought to investigate the relationship between smartphone addiction and sociodemographic characteristics, smartphone use variables, depression, anxiety, and social support. Furthermore, it aimed to identify the predictors of smartphone addiction among twelfth-grade students.

## 2 Materials and methods

### 2.1 Study design and sampling

This study was conducted according to Critical Appraisal Skills Programme (CASP) checklist (37). The research was a descriptive cross-sectional survey conducted from 21 February to 1 May 2025. It included all Palestinian twelfth-grade students in the West Bank and Jerusalem and both male and female students aged 18 years old. There were 52,142 students in the academic year 2024/2025 in the West Bank, according to the Palestinian Central Bureau of Statistics (38). Using a 0.05 level of significance, a 95% confidence level, and a 3% accuracy, the study utilized SurveyMonkey to calculate the following sample size of 1,046 students:


$$\text{Sample size}=\frac{\frac{z^{2}\times p(1-p)}{e^{2}}}{1+(\frac{z^{2}\times p(1-p)}{e^{2}N})}$$

- N = population
- e = margin of error (percentage in decimal form)
- z = z-score* (how many standard deviations data is from the mean)
*95% confidence level is a 1.96 z-score

The participants were chosen by convenience sampling, a non-probability method that selects individuals from the target population based on their accessibility. We employed an anonymous online self-administered survey approach for data collection. Researchers sent out an electronic version of the survey along with an invitation to participate by Google Forms because of travel restrictions in the West Bank and the Israeli military’s blockade of Palestinian cities. Researchers used online student groups on Facebook, other social media platforms, and WhatsApp to disseminate the survey and recruit participants. Further, we asked that teachers share the research URL with their students. A total of 1,083 students filled out the survey.

### 2.2 Tool and measures

Participants in this study completed a self-reported questionnaire consisting of three components. Section one comprised a socio-demographic sheet that collected information about the participant’s gender, grade point average (GPA), place of residence, father’s and mother’s education, and occupation. The section also included questions regarding the students’ social relationships with their father, mother, and teachers. We added additional smartphone usage-related questions, such as “Does your smartphone keep you awake at night?” Is your smartphone influencing your studies negatively? Does your smartphone affect the timing of your sleep? When did you begin using smartphones? How many hours a day do you use your smartphone? How many times each day do you check your smartphone? Do you have access to the internet? We also included questions about their overall sleep hours and exercise routine.

The second section had the Hospital Anxiety and Depression Scale (HADS), which is a 14-item scale created to assess the presence of anxiety and depression. The HADS creates two scales to distinguish the two states: HADS–A for anxiety (seven questions) and HADS–D for depression (seven questions). On a 4-point severity scale, items are rated, and each question is scored between 0 (no impairment) and 3 (severe impairment), with 3 denoting the highest anxiety or depression level. A case is considered conclusive if the score on either scale is greater than or equal to 11. A score of 0–7 is considered normal, 8–10 indicates mild depression, 11–14 indicates moderate depression, and a score of 15–21 is equal to severe depression. The internal consistency coefficient (Cronbach’s α) was 0.825. The third section had the Smartphone Addiction Scale (SPAS), which consists of a 10-item questionnaire evaluated on a 6-point Likert scale, from “Strongly Disagree” to “Strongly Agree,” with overall scores varying from 10 to 60. The current study estimated the cutoff point at 32. The internal consistency coefficient (Cronbach’s α) was 0.90. The fourth section had the Oslo Social Support Scale, which consisted of three questions. It covers different fields of social support by measuring the number of people the respondent feels close to, the interest and concern shown by others, and the ease of obtaining practical help from them. Each item is scored on a 4- or 5-point (1–4 and 1–5) rating scale. Total scores range from 3 to 14, with higher scores indicating a greater level of support. A score of 3–8 on the OSS-3 indicates poor support, 9–11 indicates moderate support, and 12–14 indicates strong support. The internal consistency coefficient (Cronbach’s α) was 0.70.

A committee of five mental health professionals evaluated the scale’s contents to confirm its cultural appropriateness, and no modifications were made. The scale was initially translated into Arabic by the study team and subsequently back-translated into English by a certified translator. During the pilot phase, we gave the tool to 25 students to assess language clarity; both the original English questionnaire and the back-translated version were examined to verify translation accuracy.

### 2.3 Data analysis

The data were analyzed with SPSS version 25 (IBM Corp., Chicago, IL, USA). The descriptive analysis for all study variables is reported in the form of frequencies and percentages, and a chi-square test was performed. Furthermore, a multivariate regression analysis was carried out, and the results were given as an adjusted odds ratio (AOR) with a 95% confidence range. The adjusted model included all potential study confounders as well as factors associated with smartphone addiction. A p-value of less than 0.05 was considered a significant association.

### 2.4 Ethical approval and consent to participate

This study adhered to the Declaration of Helsinki in all its methods. The study was approved by the Al Quds University Research Ethics Committee (Ref No: 431/REC/2024). This online survey was anonymous. At the beginning of the study, we provided written information about the aim of the study and how the data would be used. Upon filling out the questionnaire, students provided informed consent for participation in this study.

## 3 Results

### 3.1 Socio-demographic characteristics of the participants

**Table 1** indicates that the majority of participants were female (67.8%), 50.3% had a GPA ranging from 80 to 100, 48.6% resided in cities, 62.6% of participants’ fathers had less than or equal to 12 years of education, and 53.8% of participants’ mothers had less than or equal to 12 years of education. Furthermore, 66% of the respondents reported that their mothers were unemployed.

**Table 1:** Socio-demographic characteristics of participants.

Demographic characteristics		N	%
Gender	Male	349	32.2%
	Female	734	67.8%
What is your grade point average (GPA)?	Accepted (50 to 70)	247	22.8%
	Good (71 to 80)	291	26.9%
	Very good (81 to 90)	351	32.4%
	Excellent (91 to 100)	194	17.9%
Place of residency	City	526	48.6%
	Village	483	44.6%
	Camp Refugee	74	6.8%
Father’s work	Unemployed	156	14.4%
	Employed	248	22.9%
	Private work	400	36.9%
	Workers	279	25.8%
Mother’s work	Unemployed	715	66.0%
	Employed	263	24.3%
	Private work	105	9.7%
Father’s education level	>12 years	405	37.4%
	≤12 years	678	62.6%
Mother’s education level	>12 years	500	46.2%
	≤12 years	583	53.8%

### 3.2 Smartphone usage and social relationship variables

The results in **Table 2** indicate that 62.2% and 77.5% had a strong relationship with their fathers and mothers, respectively. Also, 69.5% stated that their relationship with their teachers was weak or moderate. Additionally, 47.9% indicated that they sometimes engaged in exercises, and 50.9% indicated that they slept between 7 and 9 hours each day. Furthermore, 52.2% revealed that their smartphone affected their sleep at night, 66.9% indicated that smartphone usage adversely impacts their academic work, and 57.6% stated that their smartphone usage prevents them from sleeping until midnight or later. Furthermore, 61.6% utilized their smartphones for 4 hours and over daily, and 44.5% checked their devices every 30 minutes or less.

**Table 2:** Smartphone usage and social relationship variables.

Smartphone usage and social relationship questions		N	%
How would you evaluate your relationship with your father?	Weak	100	9.2%
	Moderate	309	28.5%
	Strong	674	62.2%
How would you evaluate your relationship with your mother?	Weak	51	4.7%
	Moderate	193	17.8%
	Strong	839	77.5%
How would you evaluate your relationship with your teachers?	Weak	190	17.5%
	Moderate	563	52.0%
	Strong	330	30.5%
What are your exercise routines?	I do not do exercises	461	42.6%
	I do exercises sometimes	519	47.9%
	I do exercises frequently	103	9.5%
Total hours of sleep per day	less than and equal to 6 hours	462	42.7%
	7–9 hours	551	50.9%
	≥ 10 hours	70	6.5%
Does your smartphone keep you awake at night?	Yes	565	52.2%
	No	518	47.8%
Does the use of your smartphone affect your study negatively?	Yes	725	66.9%
	No	358	33.1%
Does your smartphone impact the timing of your sleep?	It does not affect my sleep time	460	42.4%
	It keeps me awake until 12 midnight	345	31.9%
	It keeps me awake after 12 midnight	278	25.7%
When did you start using smartphones?	Less than one year	107	9.9%
	1 year - 3 years	239	22.1%
	> 3 years	737	68.1%
How many hours do you spend using your smartphone each day?	< 2 hours	138	12.7%
	2–3 hours	278	25.7%
	≥ 4 hours	667	61.6%
How often do you check your smartphone daily?	Every 5 minutes	128	11.8%
	Every 10 minutes	148	13.7%
	Every 30 minutes	206	19.0%
	Once per hour	154	14.2%
	Every 2 to 3 hours	126	11.6%
	Many times per day	321	29.6%
Do you have access to the internet?	limited access	231	21.3%
	Always available	852	78.7%

### 3.3 Prevalence of smartphone addiction, depression, anxiety and social support

**Table 3** reveals that 57.3% of respondents exhibited smartphone addiction, 27.3% had symptoms of depression, 50.5% experienced anxiety symptoms, and 82% reported poor and weak social support.

**Table 3:** Prevalence of smartphone addiction, depress ion, anxiety and social support.

Scales		N	%
Internet addiction	<32	462	42.7%
	≥32	621	57.3%
HADs Depression scale	≤10	787	72.7%
	≥11	296	27.3%
HADs Anxiety scale	≤10	536	49.5%
	≥11	547	50.5%
Oslo Scale Social Support Scale	Poor social support	444	41.0%
	Weak social support	448	41.4%
	Strong social support	191	17.6%

### 3.4 The association between smartphone addiction and sociodemographic variables, smartphone usage variables, depression, anxiety, and social support

The Chi-square test examined the relationships between smartphone addiction, respondent characteristics, and all research variables as seen in **Table 4**. The findings indicated significant relationships between smartphone addiction and the following factors: the relationship with their father (weak, 75%, p-value < 0.001), mothers (weak, 72.5%, p-value < 0.001), and teachers (weak, 71.6%, p-value < 0.001); exercise routine (did not exercise, 63.8%, p-value < 0.001); smartphone usage that disrupted sleep at night (yes, 75.4%, p-value < 0.001); negative impact of smartphone on academic performance (yes, 71.4%, p-value < 0.001); smartphone affecting sleep duration (kept them awake after midnight, 79.7%, p-value < 0.001); duration of smartphone usage (more than 3 years, 61.6%, p-value < 0.001); daily smartphone usage hours (more than 3 hours, 70.6%, p-value < 0.001); and frequency of checking their smartphone (every 5 minutes, 80.5%, p-value < 0.001). Moreover, there were significant associations between smartphone addiction and internet access (always available, 59.5%, p-value = .006), GPA (71-80, 61.9%, p-value <.036), depression (score of 11 or higher, 68.6%, p-value < 0.001), anxiety (score of 11 or higher, 64.9%, p-value < 0.001), and social support (poor social support, 67.1%, p-value < 0.001).

**Table 4:** The bivariate correlations between smartphone addiction, and sociodemographic variables, smartphone usage variables, depression, anxiety, and social support.

Characteristics		Smartphone addiction				Chi- square
		<32		>=32		
		N	%	N	N %	P value
Gender	Male	146	41.8%	203	58.2%	.705
	Female	316	43.1%	418	56.9%	
Place of residency	City	229	43.5%	297	56.5%	.745
	Village	200	41.4%	283	58.6%	
	Camp Refugee	33	44.6%	41	55.4%	
Father’s work	Unemployed	60	38.5%	96	61.5%	.335
	Employed	98	39.5%	150	60.5%	
	Private work	178	44.5%	222	55.5%	
	Workers	126	45.2%	153	54.8%	
Mother’s work	Unemployed	304	42.5%	411	57.5%	.653
	Employed	109	41.4%	154	58.6%	
	Private work	49	46.7%	56	53.3%	
Father’s education level	>12 years	169	41.7%	236	58.3%	.632
	≤12 years	293	43.2%	385	56.8%	
Mother’s education level	>12 years	214	42.8%	286	57.2%	.931
	≤12 years	248	42.5%	335	57.5%	
How would you evaluate your relationship with your father	Weak	25	25.0%	75	75.0%	< 0.001
	Moderate	110	35.6%	199	64.4%	
	Strong	327	48.5%	347	51.5%	
How would you evaluate your relationship with your mother?	Weak	14	27.5%	37	72.5%	< 0.001
	Moderate	56	29.0%	137	71.0%	
	Strong	392	46.7%	447	53.3%	
How would you evaluate your relationship with your teachers?	Weak	54	28.4%	136	71.6%	< 0.001
	Moderate	227	40.3%	336	59.7%	
	Strong	181	54.8%	149	45.2%	
What are your exercise routines?	I do not do exercises	167	36.2%	294	63.8%	< 0.001
	I do exercises sometimes	234	45.1%	285	54.9%	
	I do exercises frequently	61	59.2%	42	40.8%	
Total hours of sleep per day	≤6 hours	207	44.8%	255	55.2%	.295
	7–9 hours	230	41.7%	321	58.3%	
	≥ 10 hours	25	35.7%	45	64.3%	
Does your smartphone keep you awake at night?	Yes	139	24.6%	426	75.4%	< 0.001
	No	323	62.4%	195	37.6%	
Does the use of your smartphone affect your study negatively?	Yes	207	28.6%	518	71.4%	< 0.001
	No	255	71.2%	103	28.8%	
Does your smartphone impact the timing of your sleep?	It does not affect my sleep time	292	63.5%	168	36.5%	< 0.001
	It keeps me awake till 12 midnight.	114	33.0%	231	67.0%	
	It keeps me awake after 12 midnight	56	20.1%	222	79.9%	
When did you start using smartphones?	<1 year	69	64.5%	38	35.5%	< 0.001
	1 year - 3 years	110	46.0%	129	54.0%	
	> 3 years	283	38.4%	454	61.6%	
How many hours do you spend using your smartphone each day?	< 2 hours	108	78.3%	30	21.7%	< 0.001
	2–3 hours	158	56.8%	120	43.2%	
	≥ 4 hours	196	29.4%	471	70.6%	
	Every 5 minutes	25	19.5%	103	80.5%	< 0.001.
	Every 10 minutes	44	29.7%	104	70.3%	
	Every 30 minutes	75	36.4%	131	63.6%	
	Once per hour	73	47.4%	81	52.6%	
	Every 2 to 3 hours	89	70.6%	37	29.4%	
	Many times per day	156	48.6%	165	51.4%	
Do you have access to the internet?	limited access	117	50.6%	114	49.4%	.006
	Always available	345	40.5%	507	59.5%	
What is your grade point average (GPA)?	Accepted (50 to 70)	96	38.9%	151	61.1%	.036
	Good (71 to 80)	111	38.1%	180	61.9%	
	Very good (80 to 90)	159	45.3%	192	54.7%	
	Excellent (91 to 100)	96	49.5%	98	50.5%	
HADs Depression scale	≤10	369	46.9%	418	53.1%	< 0.001
	≥11	93	31.4%	203	68.6%	
HADs Anxiety scale	≤10	270	50.4%	266	49.6%	< 0.001
	≥11	192	35.1%	355	64.9%	
Oslo Social Support Scale	Poor social support	146	32.9%	298	67.1%	< 0.001
	Weak social support	193	43.1%	255	56.9%	
	Strong social support	123	64.4%	68	35.6%	

Significant at p-value:< 0.05.

### 3.5 Multivariate logistic regression for determinants of smartphone addiction

We used multivariate logistic regression to examine the predictors of smartphone addiction, as shown in **Table 5**. The results indicated that students with weak (AOR: 2.854, p < 0.001) and moderate relationships (AOR: 1.818, p = 0.001) with their teacher had a significantly higher probability of smartphone addiction compared to those with strong relationships. Students who reported that their smartphone disrupted their sleep were twice as likely to develop smartphone addiction compared to those who did not (AOR: 2.143, p < 0.001). Additionally, students who indicated that smartphone usage negatively impacted their studies were three times more likely to develop smartphone addiction than those who did not (AOR: 3.006, p < 0.001). Moreover, participants who reported that smartphone usage did not influence their sleep time (AOR:.478, p value = 0.001) were less likely to develop smartphone addiction compared to those who indicated it disrupted their sleep after midnight. Moreover, participants who used smartphones for less than 2 hours daily (AOR: 0.347, p < 0.001) and those who used smartphones for 2 to 3 hours daily (AOR: 0.684, p = 0.037) displayed a lower likelihood of developing smartphone addiction compared to participants who engaged with smartphones for 4 hours and over daily. Finally, participants indicating poor social support (AOR: 3.051, p < 0.001) or weak social support (AOR: 2.127, p < 0.001) had a higher likelihood of developing smartphone addiction compared to those with strong social support. Depression and anxiety were not a predictor of smartphone addiction.

**Table 5:** Multivariate logistic regression for determinants of smartphone addiction.

Characteristics	Smartphone addiction			Adjusted analysis 95% CI, AOR*	
		Sig.	AOR	Lower	Upper
How would you evaluate your relationship with your teachers?	Weak	. < 0.001	2.854	1.777	4.583
	Moderate	.001	1.818	1.292	2.557
	Strong	References			
Does your smartphone keep you awake at night?	Yes	. < 0.001	2.143	1.520	3.021
	No	References			
Does the use of your smartphone affect your study negatively?	Yes	. < 0.001	3.006	2.153	4.197
	No	References			
Does your smartphone impact the timing of your sleep?	It does not affect my sleep time	.001	.478	.307	.745
	It keeps me awake till 12 midnight	.528	.874	.575	1.328
	It keeps me awake after 12 midnight	References			
How many hours do you spend using your smartphone each day?	< 2 hours	. < 0.001	.347	.205	.586
	2–3 hours	.037	.684	.479	.977
	≥ 4 hours	References			
How often do you check your smartphone daily?	Every 5 minutes	.003	2.399	1.359	4.237
	Every 10 minutes	.136	1.446	.890	2.350
	Every 30 minutes	.085	1.451	.949	2.217
	Once per hour	.289	1.287	.807	2.051
	Every 2 to 3 hours	.055	.594	.350	1.011
	Many times per day	References			
Oslo Social Support Scale	Poor social support	. < 0.001	3.051	1.964	4.742
	Weak social support	. < 0.001	2.127	1.393	3.249
	Strong social support	References			

The logistic regression model was adjusted for all study variables.

Significant at p-value: < 0.05.

## 4 Discussion

Younger generations may be more inclined to adopt new smartphone technologies, thus increasing addiction risk (39). This study investigated smartphone addiction among 12th-grade students. The findings revealed that 57.3% of participants experienced smartphone addiction. This finding is higher than those of previous studies and exceeds the rate of smartphone addiction in young adult populations internationally, which ranges from 30% to 45% (15). A South Korean research study found that 30.9% of middle school children were at risk of smartphone addiction, while 69.1% were regular users (40). Kim and Baik reported that 15.9% of high school students had smartphone addiction (41). Another survey indicated that 24.7% of high school students were smartphone addicts, while 75.3% were regular users (42). According to Çağan and Koca (43), 36.9% of Turkish high school students who use smartphones experienced addiction (43). In addition, a Chinese study indicated that 22.3% of junior high girls and 23.2% of boys had smartphone addiction (44). Also, one study found that 35.9% of Thai students and 12% of Japanese students are addicted to their smartphones (45). In a study by Haug et al., 16.9% of Swiss vocational school students reported smartphone addiction (10). One possible explanation of high prevalence of smartphone addiction in the current study is that, as a result of the ongoing occupation and political violence in Palestine, adolescents may be more likely to use their smartphones to cope with stress, depression, and trauma, potentially increasing their risk of smartphone addiction. Psychological trauma is a well-examined factor that increases the risk of developing addiction disorders (46). Recent studies found that the prevalence of PTSD symptoms among Palestinian youth was 65.9% (47) and 65.9% experienced depression, and 60.9% experienced anxiety during the current 7 October war (27). According to Lawson et al., individuals who have experienced trauma are more likely to acquire a particular addiction disorder than the general population (48). This high prevalence in the current study may indicate major health concerns in this age group in Palestine. Smartphone addiction is associated with reduced work productivity and academic performance (49, 50), emphasizing the harmful influence on these students’ future lives and careers. Additionally, smartphone addiction may exacerbate obesity by encouraging inactivity and increasing the risk of traffic-related accidents and fatalities (51, 52).

The elevated prevalence rate observed in this study signifies a possible public health issue associated with smartphone usage among high school students. A preventative campaign is needed to promote awareness among students, families, instructors, and Ministry of Education policymakers about the health and academic problems associated with smartphone addiction among high school students. The results indicated that students with weak and moderate relationships with their teacher had a significantly higher probability of smartphone addiction compared to those with strong relationships. This finding is similar to other studies (53, 54). Research indicates that excessive smartphone usage adversely impacts social relationships (8, 12, 12, 52, 55). Choi et al. found that smartphone addiction adversely impacted interpersonal interactions and mental health (55). One study revealed that one-third of the participants favored initiating conversation using smartphones or social media, exhibiting considerably higher addiction levels (8). Celikkalp et al. showed that the participants’ communication ability scores diminished as their addiction scores escalated (8). This finding may underscore the necessity of enhancing the relationship between teachers and students, particularly as students experiencing high stress may resort to smartphones for communication, resolving academic tasks, and obtaining information rather than consulting their teachers, potentially exacerbating smartphone addiction. Supportive teacher-student connections were shown to facilitate active student participation in school activities and enhance school engagement (56, 57).

Moreover, students who reported that their smartphone disturbed their sleep were twice as likely to develop smartphone addiction compared to those who did not, a finding supported by previous research (58–62). Excessive smartphone usage has been linked to daytime fatigue, prolonged sleep latency, and reduced sleep duration (62, 63). Moreover, excessive nighttime smartphone usage may prolong wakefulness, hence compromising sleep quality (43). There are several studies that have shown the negative effects of smartphone use on sleep, including a decrease in melatonin hormone release as a result of prolonged exposure to screen light, a delay in the circadian rhythm, and an increase in curiosity as a result of social media use through smartphones, which in turn leads to trouble falling asleep and disrupts sleep (64, 65). This finding may indicate the potential dangers of smartphone addiction for the health of adolescents in their final year of high school. Therefore, students need to be made more aware of the correlation between smartphone addiction and sleep problems through an awareness campaign. To understand the connection between smartphone addiction and sleeplessness, additional study is required. In addition, students who said their smartphone use had no effect on their sleep time were less likely to develop an addiction to their device than those who said it kept them up after midnight. According to a study conducted by Cha and Seo, those who played games between 4:00 and 8:00 p.m. after school were 2.65 times more likely to have a smartphone addiction than those who played apps first thing in the morning (40). Furthermore, using a smartphone after 1 a.m. increased the chance of addiction by a likelihood of three (15). This association may indicate a lack of self-control and the persistence of use despite negative consequences, which are hallmarks of behavioral addiction (15). Previous research on teens found that having a smartphone was associated with more late-night and early-morning social media use (66).

In this study, students who used smartphones for less than 2 hours a day, as well as those who used them for 2–3 hours a day, were less likely to be addicted to smartphones than those who used them for 4 hours or more each day. For instance, Çağan & Koca (43) similarly found that the frequency of smartphone addiction among students escalated with an increase in their daily smartphone screen time. They found that everyday smartphone usage for 5 hours or more elevates the likelihood of smartphone addiction (43). Furthermore, Cha and Seo identified that those who were at risk for smartphone addiction comprised students who utilized a smartphone for an average of 313.13 minutes daily (40). However, Sohn et al. (15) argued that while heavy use is seen in people with any addiction, just using a lot isn’t enough to prove someone is addicted, based on the ICD-11 criteria for gaming and gambling disorders (15, 67). For addiction to exist, subjective suffering and functional impairment must also be present. Some individuals may exhibit addictive characteristics after a brief period of usage, whereas others may utilize their phones adaptively over extended durations yet can disengage from their devices without distress and engage in suitable activities, such as interacting with family members or adhering to a timely bedtime (68).

Further, our data suggested that students who reported that smartphone usage adversely affected their academic performance were three times more likely to develop smartphone addiction compared to those who did not. Other studies corroborated this finding (38, 39). Studies have linked excessive smartphone usage to diminished academic performance (8). Celikkalp et al. found that the academic scores of students who persisted in using smartphones during class were considerably lower (8). Cho and Lee reported that smartphone usage adversely impacted classroom learning, and it was advised that regulations and procedures be established to manage smartphone use in educational settings (26). Research indicates that phone usage in the classroom is inappropriate, since it distracts both the user and others in the surroundings, leading to complaints (26, 51, 69). Conversely, Yıldırım et al. contended that the educational potential of smartphones was overlooked and that these devices may enhance teaching and learning if a suitable technological and contextual framework were established (70). Therefore, regulating students’ smartphone usage is essential to prevent detrimental effects on their academic performance and to improve their concentration on studies.

Finally, our study found that students who had poor or weak social support were more likely to develop smartphone addiction than those with strong social support, which is consistent with previous studies (71, 72). Individuals who engage in various activities, such as online socializing and seeking pleasure and knowledge via smartphone, may get positive social support (73). Several studies have found a negative relationship between smartphone addiction and social support (74, 75), implying that lower social support is related to greater severity of internet addiction (76). Herrero et al. showed that social support predicted smartphone addiction and argued that certain user traits, such as extroversion, neuroticism, and sensation-seeking, may influence the emergence of social support associated with smartphone addiction (77). According to Aksoy (78), the primary motivation for using social media was a lack of friendships. As a result, when people feel a lack of social support in their lives, such as respect or help from significant others, they may turn to technology, particularly smartphones, which serve as easily accessible communication tools with strong social features, allowing for compensatory social interaction (78). Mobile phone communication helps people to maintain acceptable interpersonal relationships and seek assistance from others. Nevertheless, it may lead to smartphone addiction (79). Our findings suggest that increasing social support from students’ parents, teachers, and peers can help reduce mobile phone addictions. Zhong et al. revealed that strong social support can reduce negative emotions and increase self-control, allowing for better smartphone usage management (80).

There were several limitations to this study. Cause-and-effect links cannot be inferred since data collection is cross-sectional. Furthermore, the self-reported data collection approach we utilized may have introduced biases. Because a convenience sample approach was employed, the prevalence estimate may be interpreted with caution. Furthermore, one may exercise caution when generalizing the findings of this study, given that the sampled group does not accurately represent the 12th-grade students throughout Palestine.

### 4.1 Implication for practice

For the Palestinian community to undergo an information-centric transformation, the involvement of young people with new technologies is vital. Excessive smartphone usage negatively impacts the health, education, and well-being of individuals and society. So, to help adolescents’ mental health and communication skills, we may need to adopt interpersonal, community and family based approaches to help students limit how much time they spend on smartphones. These results are worth being considered by public and mental health professionals when they produce recommendations for initiatives aimed at preventing and controlling smartphone addiction and teen smartphone usage. Therapists, parents, and teachers are all advised to consider how much time spent on mobile devices affects students’ ability to sleep and how well they perform in school. The results suggest that the length of time spent using a smartphone and the last time it was used might be indicators of susceptibility to smartphone addiction. Students may need training on how to manage their smartphone addiction if they use their phones for four hours or more every day or after midnight. To provide these students with a nurturing home life, a strong sense of family connection, and emotional support, it is essential that parents and guardians communicate openly with one another. Involvement in extracurricular activities, participation in cultural and recreational events, development of interpersonal and communication skills, and provision of social assistance are all areas in which schools aim to engage their students. To help students overcome incorrect thinking, make better use of social support, and limit their smartphone usage, we may take an active approach. The long-term effects of smartphone use on health, including addiction and sleep disruption, can be the focus of future studies. Investigating the factors that may contribute to smartphone addiction among students in the 12th grade may include stress, personality traits, mental illness, political violence, and expectations from both families and schools. In addition to longitudinal quantitative research, qualitative studies examining the connection between social support and smartphone addiction are necessary.

## 5 Conclusion

Smartphone addiction was prevalent in high school students in this study. Weak teacher relationships, sleep disturbances, academic negative effects, and insufficient social support may lead to smartphone addiction. Programs that educate students, parents, and educators on smartphone addiction can prevent it and help detect and manage smartphone use problems.

## Data Availability

The original contributions presented in the study are included in the article/Supplementary Material. Further inquiries can be directed to the corresponding author.
